# Individually Modified Microneedle Array for Minimal Invasive Multi-Electrolyte Monitoring

**DOI:** 10.3390/bios15050310

**Published:** 2025-05-12

**Authors:** Ketian Yu, Yukun Ma, Yiming Wei, Wanying Chen, Zhen Dai, Yu Cai, Xuesong Ye, Bo Liang

**Affiliations:** 1 Biosensor National Special Laboratory, Key Laboratory of Biomedical Engineering of Ministry of Education, College of Biomedical Engineering and Instrument Science, Zhejiang University, Hangzhou 310027, China; 3220101305@zju.edu.cn (K.Y.); 22260304@zju.edu.cn (Y.M.); 22415049@zju.edu.cn (Y.W.); 22215041@zju.edu.cn (W.C.); 22215043@zju.edu.cn (Z.D.); yexuesong@zju.edu.cn (X.Y.); 2 Binjiang Institute of Zhejiang University, Hangzhou 310053, China; 0623873@zju.edu.cn

**Keywords:** electrochemical biosensor, microneedle electrode array, ion-selective electrode, minimally invasive monitoring

## Abstract

Electrolytes play crucial roles in regulating nerve and muscle functions. Currently, microneedle technology enables real-time electrolyte monitoring through minimally invasive methods. However, due to the small size of microneedles, performing multi-layer modifications on individual microneedles and ensuring the integrity of these layers pose significant challenges. Additionally, the puncture efficiency of the electrodes will be affected by the structure of microneedle array integration. To address these issues, we primarily focus on developing a multi-parameter ion monitoring system based on microneedle arrays. By optimizing the surface reconstruction of electrode substrates, the adhesion between the electrode surface and the modification layer was improved, enhancing the stability of the electrodes. Potassium, sodium, and calcium ion-selective electrodes based on microneedles were fabricated, demonstrating good sensitivity and linearity. To tackle the puncture efficiency of microneedle arrays, finite element simulation was employed to investigate the mechanical properties of different structural designs of microneedle arrays during skin insertion. Ultimately, an integrated microneedle array was designed and assembled, and a multi-parameter ion monitoring system was developed, validated through in vitro simulations and in vivo animal experiments. This research provides valuable insights into the development and advancement of minimally invasive, multi-parameter dynamic monitoring technologies in clinical settings.

## 1. Introduction

Electrolytes are essential substances for maintaining normal physiological functions in the human body, and their dynamic concentration changes are significant indicators of health status [1,2]. The real-time, convenient, and accurate monitoring of electrolyte levels holds substantial application value in clinical diagnostics, disease management, and sports health monitoring [3,4,5]. Biochemical sensors combine electrolyte ion detection technology with biochemical sensing techniques to dynamically monitor electrolyte ions in body fluids using non-invasive or minimally invasive methods, providing real-time insights into health conditions with high accuracy, convenience, and lower costs [6,7,8].

When applied to biochemical sensors, microneedle technology typically involves modifying the microneedle surface with specific membrane layers to achieve the desired detection capabilities [9,10,11]. Xiu et al. proposed a highly integrated wearable solid contact-ISE combined with the hardware part through employing a printed circuit board substrate, allowing multiple measurements with repeatable open circuit potential, achieved by applying a positive potential on the working electrode [12]. The Parrilla team designed an all-solid-state microneedle sensor for real-time potassium ion monitoring, which is used for the clinical analysis of interstitial fluid potassium ions [7]. It can puncture the skin’s stratum corneum to reach the interstitial fluid layer without causing pain. Zhang et al. developed a multifunctional microneedle sensing patch designed for the rapid healing of bacterially infected wounds and visual monitoring of wound pH, establishing a strategy that combines therapy and sensing to address delayed wound management [13]. Gowers et al. fabricated microneedle array substrates using polymer materials through laser ablation technology and subsequently coated the polymer substrate surfaces with conductive metal layers [14]. Xu et al. developed a fully integrated microneedle array for fitness-related biomarker monitoring, demonstrating the 3D-printed biocompatible microneedles can painlessly penetrate the stratum corneum and access interstitial fluid for analysis [15].

As an emerging biosensing tool, microneedle technology can directly obtain real-time concentration information of electrolytes in subcutaneous tissue fluid using minimally invasive methods, offering a more efficient, precise, and convenient solution for electrolyte monitoring [16,17,18,19]. However, several challenges and issues remain in practical applications [9,20,21]. First, due to the small size of microneedles [22], performing multi-layer fine modifications and ensuring the integrity of these modification layers pose significant challenges during microneedle fabrication [23,24,25]. Second, the design of microneedle arrays directly affects the uniformity of force distribution and the degree of skin tissue damage during puncture [26,27]. Poor design may lead to uneven force distribution, causing excessive pressure on some microneedles and increasing the risk of skin tissue and electrode damage [28]. Third, if one needle in a multi-needle array fails, it could cause the entire system to fail, increasing usage costs. Many microneedle array designs are also limited by integration processes, making it difficult to achieve good skin adhesion [25,29].

**Figure 1:**
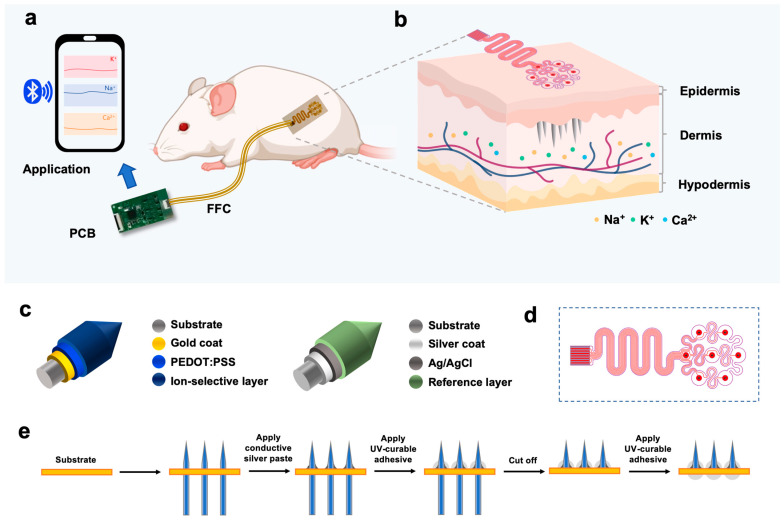
(**a**) Schematic of the minimal invasive multi-electrolyte monitoring system. Integrate the microneedle array with the PCB hardware platform, and transmit the data to the phone via Bluetooth. (**b**) Schematic of subcutaneous detection. The microneedle penetrates the epidermis to reach the interstitial fluid, allowing the simultaneous detection of Na^+^, K^+^ and Ca^2^^+^. (**c**) Individually modified multi-layer structure of microneedle for working and reference electrodes, respectively. (**d**) Flexible array design diagram. (**e**) Assemble steps of individually modified microneedle array.

This study primarily focuses on developing and constructing a multi-parameter ion monitoring system based on microneedle arrays for the real-time continuous detection of subcutaneous ions (Figure 1a,b). The research optimizes the reconstruction of microneedle electrode substrates to improve the adhesion between different modification layers [30,31]. By investigating the impact of various coating methods on membrane stability, higher-stability and -reliability ion-selective microneedle electrodes were constructed (Figure 1c). Reference electrode stability was optimized to maintain potential stability in solutions with varying chloride ion concentrations. Using Abaqus simulation, the influence of different structural designs of microneedle arrays on puncture efficiency was explored. Multiple microneedle electrodes were integrated into an array featuring serpentine wiring and hollow designs (Figure 1d,e), allowing better skin adhesion and ensuring the stable retention of microneedles within skin tissues during stretching and twisting deformations [32,33]. The study designed hardware circuits and an upper-level computer system for multi-parameter ion monitoring, enabling real-time data uploading to a mobile application via Bluetooth for real-time monitoring, storage, and analysis. The feasibility and reliability of the multi-parameter ion monitoring system were ultimately validated through in vitro simulations and in vivo animal experiments. This research provides valuable insights into the development and advancement of minimally invasive, multi-parameter dynamic monitoring technologies in clinical settings [34,35].

## 2. Materials and Methods

### 2.1. Preparation of Ion-Selective Electrodes Based on Microneedles

Surface reconstruction of microneedle electrodes. We prepared an electrode etching solution by diluting standard gold etchant (1.2924 g/mL) with DI water at a ratio of 1:10. The microneedle electrodes were then soaked in this etching solution for 120 s to achieve surface reconstruction (Figure S1).

Preparation of ion-selective electrodes. First uniformly coat the microneedle surfaces with an insulating varnish, leaving only the 1 mm tip exposed as the active area of the microneedle electrode. Using chronoamperometry at a constant current of 14 μA for 700 s in a PEDOT:PSS electroplating solution, a uniform PEDOT:PSS film was electro-polymerized onto the microneedle electrode surface (Figures S2 and S3). We used the ring coating method to modify the microneedle (Figure S5).

Preparation of reference electrodes. Silver-coated microneedles were chloridized by applying a constant voltage of 0.6 V in a 0.1 M HCl solution for 40 s. After chloridization, the electrodes were rinsed with DI water to remove residual HCl and then air-dried. Once completely dry, the chloridized microneedle electrodes were dip-coated by immersing them in a reference solution and slowly withdrawing them. The electrodes were then dried in the dark for 4 h. The prepared electrodes were stored in a 3 M KCl solution.

The preparation reagents are described in detail in Supplementary Materials S1.

### 2.2. Finite Element Modeling and Simulation and Experimental Validation

Analysis of insertion force results. The insertion force of a microneedle refers to the reaction force experienced by the microneedle during its penetration into skin tissue. This force primarily consists of rigidity force, friction force, and cutting force. From the simulation results, it is evident that during insertion, the maximum stress occurs at the tip region and around the largest cross-section of the microneedle penetrating the dermis. The stress at the tip region mainly comes from rigidity and cutting forces, while the stress acting on the cross-section is primarily due to residual rigidity and friction forces. Therefore, comparing the insertion forces of arrays with different spacings can help select the optimal array configuration.

Analysis of insertion efficiency results. Since microneedles only function below the stratum corneum in the dermis, the insertion efficiency $E_{p}$ is defined as the length of the microneedle inserted below the stratum corneum *L* relative to the total microneedle length *H*. For circular microneedle arrays, when the array punctures the skin, the center of the force distribution area experiences the greatest deformation and compression, which gradually decreases towards the edges. The insertion efficiency differs between central and edge microneedles within the array. Central microneedles, due to their symmetrical nature, have equal forces on both sides ($L_{1}$). Edge microneedles, however, experience unequal forces on either side ($L_{2}$ and $L_{3}$). When analyzing edge microneedles, the average length of both sides is used in calculations.

Analysis of skin deformation. Let $L^{\prime }$ represent the length of the microneedle inserted into the skin tissue, which also corresponds to the height of the deformed skin tissue. Define point A as the center of the wound on the skin surface, with distance $L_{1}^{\prime }$ from the needle tip. Point B is the center of the edge wound, with distance $\left(L_{2}^{\prime }+L_{3}^{\prime }\right)/2$ from the needle tip. The horizontal distance between points A and B is the microneedle spacing d. By fitting the skin wound, we can calculate the degree of skin deformation, denoted by the arc AB radius R. A smaller arc radius indicates deeper skin deformation, whereas a larger radius signifies less deformation.

Experimental validation. To validate the accuracy of the simulation results in practical applications, we conducted puncture experiments using microneedle arrays and pig skin to measure the insertion forces required for different microneedle arrays. We prepared microneedle arrays with spacings of 1.0 mm, 1.5 mm, 2.0 mm, 2.5 mm, and 3.0 mm. To prevent twisting or deformation during insertion, we used rigid printed circuit boards as substrates for the microneedle arrays. The microneedle arrays and pig skin were fixed to the upper and lower fixtures of a universal material testing machine. The fixture holding the microneedle array was slowly lowered to collect force–displacement curves. In each experiment, the position of the pig skin was adjusted after each puncture to avoid repeated insertions at the same location, which could affect the insertion force results. Finally, we compare the measured insertion forces with the simulation results to verify the accuracy of our finite element models.

### 2.3. Integration of Microneedle Arrays and Testing of the Monitoring System

Integration of microneedle arrays. Vertically place the prepared microneedle electrodes into the through-holes of the array substrate, leaving approximately 2 mm of the electrode tip exposed. Apply conductive silver paste to both the microneedles and the copper pads on the substrate to establish electrical connections. Cut off any excess electrode material below the array substrate and apply UV-curable adhesive to the cut ends (Figure 1e).

Testing the monitoring system in a working environment. We compared the performance of the monitoring system with that of an electrochemical workstation using the same microneedle array electrode. During testing, the microneedle array substrate is connected to the hardware circuit via an electrode interface. The array is then immersed in DI water, and high-concentration ion solutions are sequentially added to test the sensitivity of the microneedle electrodes using the electrochemical workstation.

### 2.4. In Vitro Simulation Experiments

To investigate the impact of penetration depth on the performance of ion-selective microneedle electrodes, we established an in vitro simulation platform based on agarose gel (Figure S15). Using an electric displacement platform, we controlled the penetration depth of the microneedle array to study the sensitivity of microneedle electrodes at different depths and to verify their performance in non-liquid environments. We secured the prepared microneedle array and agarose gel on the bases. We adjusted the electric displacement platform to make the microneedle array just touch the surface of the agarose gel, and marked this point as a penetration depth of 0. We then incrementally increased the penetration depth by 0.2 mm each time until the microneedle array was fully inserted into the agarose gel (1.2 mm). We then recorded the electrode responses at penetration depths ranging from 0.2 mm to 1.2 mm, and repeated the above steps with different concentrations of agarose gel to obtain potential responses at various ion concentrations and penetration depths. We then integrated the experimental data to derive sensitivity curves for different penetration depths.

### 2.5. In Vivo Animal Experiments

We conducted in vivo experiments using healthy SD rats purchased from Hangzhou Medical College, via the following steps: Prepare multi-parameter microneedle arrays. Gas-anesthetize the SD rats, shave most of the fur on the back of the rat and remove remaining hair with depilatory cream. Use a suitable rigid support to stabilize the flexible array, ensuring uniform and vertical penetration into the skin. Secure the microneedle array with medical dressings. Connect the monitoring circuit board to the microneedle array via an electrode interface and fix it with medical tape to prevent detachment during the experiment. Observe the electrode signals in real time using the smartphone application. Once the signals stabilize, inject 6% NaCl solution (0.5 mL/100 g) into the rat’s abdominal cavity to induce changes in electrolyte concentration. Monitor and record the fluctuations in electrolyte levels via the smartphone application. Validate the electrode test results using biochemical analysis methods. After the smartphone application signals stabilized, blood samples (0.3–0.5 mL) were collected every 15 min from the retro-orbital vein. Blood samples were centrifuged at 4000 rpm for 10 min to obtain serum. The collected serums were tested by use of a biochemical analyzer (Roche Cobas C 311 Fully Automated Biochemical Analyzer) to measure the concentrations of sodium, potassium, and calcium ions in the blood. This process provides objective biochemical detection data to accurately compare and analyze the relationship between electrode signals and actual blood electrolyte concentrations.

## 3. Results and Discussion

### 3.1. Performance Characterization of Ion-Selective Electrodes Based on Microneedles

**Figure 2:**
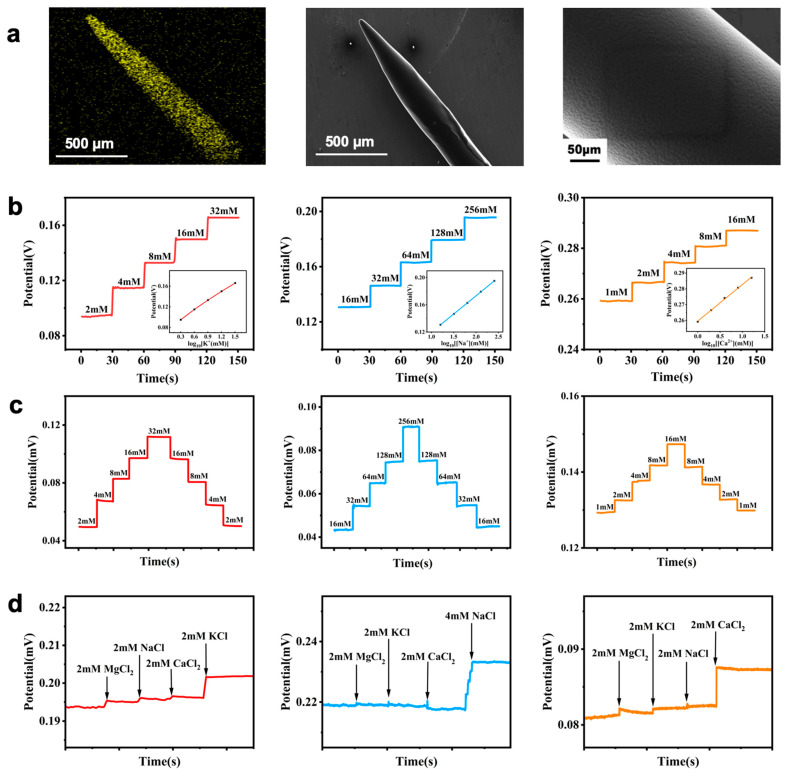
(**a**) EDS images of uniform coating of PEDOT:PSS on the reconstructed substrate of ion-sensing microneedle electrodes; SEM images of ion-sensing microneedle electrodes after coating with ion-selective membranes using the ring-coating method. Magnified SEM images showing uniform coating of the selective membrane. (**b**) Linear response of ion-sensing microneedle electrodes (K^+^, Na^+^, and Ca^2^^+^). (**c**) Reproducibility of ion-sensing microneedle electrodes (K^+^, Na^+^, and Ca^2^^+^). (**d**) Selectivity of ion-sensing microneedle electrodes (K^+^, Na^+^, and Ca^2^^+^).

In this study, gold-coated microneedle electrodes and silver-coated microneedle electrodes were used as substrates. By modifying the microneedle surfaces with multiple functional layers, ion-selective microneedle electrodes and all-solid-state reference electrodes were fabricated. First, the microneedle substrates were subjected to soaking etching, which significantly enhances their performance (Figures S2–S4). Poly(3,4-ethylenedioxythiophene): poly(styrenesulfonate) (PEDOT:PSS) was electro-polymerized at the tip of the microneedles to form an ion-to-electron conversion layer, followed by coating with an ion-selective membrane using a dip-coating method to produce reliable and robust working electrodes. The modified microneedle electrodes exhibited good sensitivity, linearity, reproducibility, selectivity (Figure 2b–d), and stability (Figure S6). Upon testing, the K^+^ selective electrode showed a sensitivity of 58.80 mV/decade and a linearity of 0.998; the Na^+^ selective electrode had a sensitivity of 53.61 mV/decade and a linearity of 0.999; the Ca^2+^ selective electrode demonstrated a sensitivity of 23.32 mV/decade and a linearity of 0.998, all close to theoretical values predicted by the Nernst equation. The tested concentration ranges covered physiological variations of potassium, sodium, and calcium ions in the human body. A highly stable reference electrode was prepared by coating the silver-coated microneedle electrodes with a chloride solution followed by a reference membrane (Figure S7). The potential change rate of the reference electrode is 0.9 mV/h. The selectivity of an ion-selective electrode refers to its specific response capacity to the target ion, meaning that in a mixed solution containing various ions, the electrode can selectively respond to the target ion while being less sensitive to the influence of other interfering ions. Figure 2d shows the test results for the ion-selective electrodes. During the potassium ion selective electrode test, the solution initially contained 2 mM KCl. After the potential signal stabilized, measurements were recorded as 2 mM MgCl_2_, 2 mM NaCl, and 2 mM CaCl_2_ were sequentially added, resulting in only minor changes in the electrode potential. Then, a high-concentration KCl solution was added, increasing the KCl concentration in the solution to 4 mM, which resulted in a significant potential change, indicating that the potassium ion electrode has good selectivity and that its sensitivity is not affected by external cations. The testing methods for the sodium ion electrode and calcium ion electrode were the same, with results shown in Figure 2d.

### 3.2. Optimization of Array Design Using Finite Element Analysis

**Figure 3:**
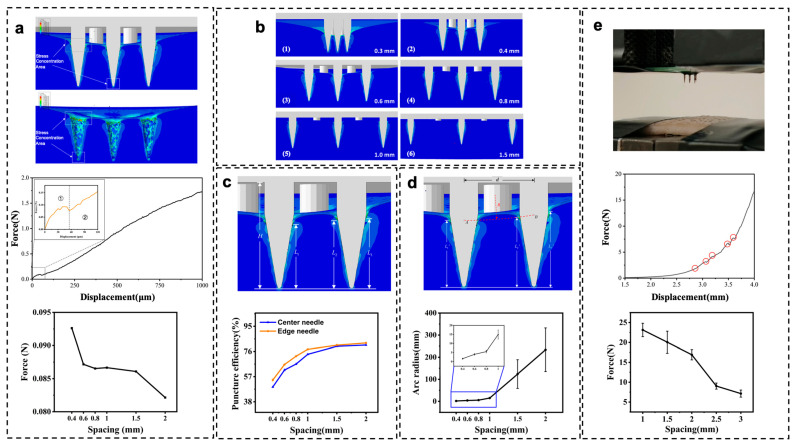
(**a**) Microneedle array insertion into skin tissue: force and local stress simulation diagram, stress–strain curve of the microneedle array’s insertion into skin tissue, ① and ② represent the stress changes before and after the puncture point, insertion force of microneedle arrays with different spacings. (**b**) Simulation results of microneedle arrays with different spacings. (**c**) Analysis of insertion efficiency of microneedle array insertion into skin tissue, puncture efficiency of central and edge microneedles in microneedle arrays with different spacings. (**d**) Analysis of skin deformation degree caused by microneedle array insertion into skin tissue, skin deformation degree after insertion of microneedle arrays with different spacings. (**e**) Puncture experiment of microneedle array on pig skin, force–displacement curve of microneedle array puncturing pig skin, insertion force magnitude of microneedle arrays with different spacings puncturing pig skin.

The design of the Finite Element Modeling Simulation is shown in Figures S8 and S9 (Supplementary Materials), and the simulation results are shown in Figure 3. The stress–strain curve during microneedle insertion is illustrated in Figure 3a. As the microneedle array contacts the skin, the skin deforms, and the stress on the microneedles gradually increases. When the force on the microneedle tips reaches the von Mises yield stress, skin units fail, indicating that the skin tissue begins to rupture. This causes a brief and rapid drop in stress (highlighted as section ① in the curve in Figure 3a), which defines the puncture point where the maximum force is recorded as the insertion force required to pierce the skin. As the microneedle array punctures further, the stress gradually increases until full insertion. Comparative studies on insertion forces for different pitch circular arrays are also shown in Figure 3a. With a pitch of 0.4 mm, the maximum insertion force is 92.62 mN. As the pitch increases, the insertion force decreases, reaching a minimum of 82.15 mN at a pitch of 2 mm. This is due to the presence of higher localized stress when the pitch is smaller, leading to increased friction between the needles and more significant insertion forces. Larger pitches distribute the force more evenly, resulting in similar forces on each needle during insertion, primarily influenced by the number of needles.

Due to skin deformation, microneedles do not fully puncture, and the insertion depth varies with different pitches. The insertion efficiency was assessed using Query tools to calculate the penetration length below the dermis (Figure 3c). For a pitch of 0.3 mm, the insertion efficiencies of center and edge needles are 41.67% and 48.23%, respectively. At a pitch of 2.0 mm, these efficiencies increase to 80.19% and 81.8%.

Skin deformation affects microneedle insertion efficiency. As shown in Figure 3d, smaller pitches (0.3 mm to 1 mm) result in smaller arc radii (within 20 mm), while larger pitches (>1 mm) lead to much larger arc radii (>100 mm), making the skin surface almost flat and minimizing visible deformation. Center and edge needles exhibit similar insertion depths, introducing larger error bars into calculations.

The finite element simulation results provide valuable insights into the mechanical behavior of microneedle arrays during skin insertion. By analyzing the insertion efficiency of different microneedle array designs, we were able to optimize the spacing between microneedles to achieve improved insertion efficiency and reduced insertion force. This optimization is crucial for the performance of the microneedle-based monitoring system, as it directly affects the contact between the microneedle electrodes and the interstitial fluid. When microneedles are inserted into the skin with higher efficiency, they penetrate more uniformly and consistently, ensuring better contact with the target analytes. This results in more reliable and accurate ion concentration measurements. The optimized design minimizes the variation in insertion depth among individual microneedles, reducing signal interference and improving the overall stability of the monitoring system.

To validate the simulation accuracy, microneedle arrays were tested on pig skin (Figure 3e). Unlike in the simulations, multiple insertion points were observed due to skin twisting and varying needle heights. The last insertion point’s force was recorded as the insertion force. Testing three times for each array revealed discrepancies from simulations due to differences between pig and human skin properties. However, the trend of decreasing insertion force with increasing pitch was consistent, supporting previous conclusions. The simulation results guided the experimental validation, where microneedle arrays with optimized spacing demonstrated good performance in experiments. These results highlight the importance of structural design in microneedle array.

### 3.3. Design of Monitoring System Based on Microneedle Arrays

During sensor use, skin deformation due to squeezing, stretching, bending, or twisting can cause friction or detachment of microneedle arrays. Therefore, serpentine wiring and hollow array designs ensure better skin adhesion and flexibility. PDMS (Polydimethylsiloxane) was used to simulate skin. The microneedle arrays were fully inserted into the PDMS, and then subjected to stretching and twisting tests (Figure S10). The results show that the array substrate could deform along with the PDMS, while the microneedles remained securely embedded within the PDMS without detaching (Figure S10). Testing before and after electrode assembly confirmed that there was no difference in electrode sensitivity (Figure S11).

**Figure 4:**
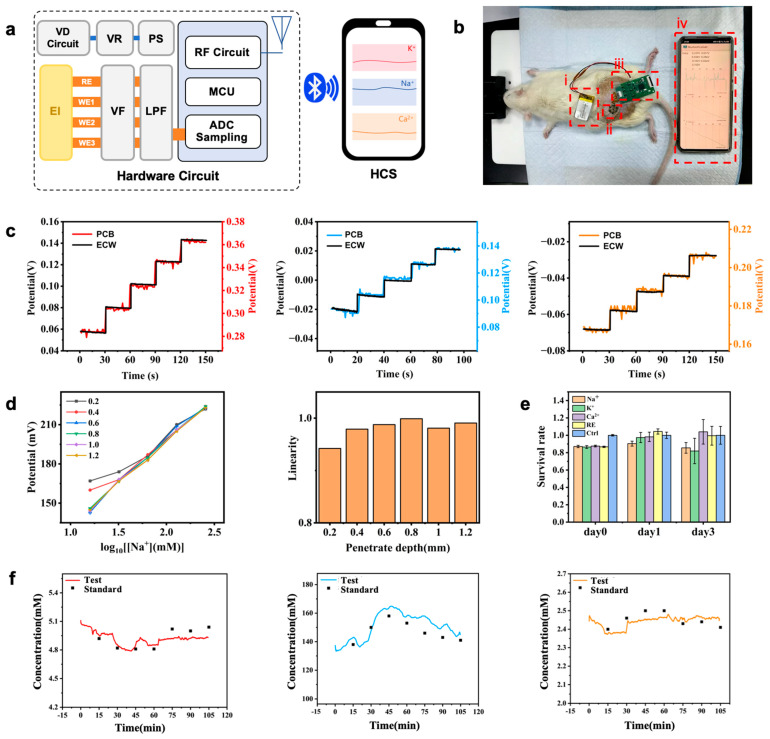
(**a**) Monitoring system architecture (VD Circuit—voltage divider circuit; EI: electrode interface). (**b**) In vivo animal experiment scenario, (i) power supply, (ii) microneedle array, (iii) monitoring circuit, (iv) mobile phone application. (**c**) Monitoring system working environment test results (K^+^, Na^+^, and Ca^2^^+^). (**d**) Electrode sensitivity curves and their linearity at different insertion depths. (**e**) Cell Counting Kit-8 (CCK-8) results of the microneedle electrodes. (**f**) Comparison of concentration values corresponding to monitoring system-collected data with standard concentrations (K^+^, Na^+^, and Ca^2^^+^).

A complete monitoring system was developed (Figure 4a), including hardware circuits (Figure S12) and software systems (Figures S13 and S14). Electrode interfaces connect to microneedle arrays; power circuits provide stable voltage; detection circuits transmit potential signals to the ADC module in the main control chip, converting them into digital signals for Bluetooth transmission (Figure S12). The low-power Bluetooth system communicates with a smartphone application, enabling real-time data reception, visualization, and storage.

System performance was comprehensively tested, including the basic performance of both the hardware circuits and software systems as well as the reliability of the monitoring system in actual application environments (Figure 4c). The results were consistent with those obtained from the electrochemical workstation using the same microneedle array, indicating that the monitoring circuit has reliable testing capabilities in practical applications and can accurately reflect the performance of the microneedle array.

### 3.4. In Vitro Simulation Experiments

In vitro simulation experiments were conducted using an electric displacement platform to control the penetration depth of microneedle arrays. This allowed us to study the sensitivity of microneedle electrodes at different penetration depths and to validate their performance in non-liquid environments. By controlling the penetration depth, we obtained potential responses of microneedle electrodes at various depths for each ion concentration. After data consolidation, we derived sensitivity curves for different penetration depths and analyzed their linearity, as shown in Figure 4d. The sensitivity linear equations measured at depths of 0.2~0.4 mm showed some deviations compared to other conditions, while for depths greater than 0.6 mm, the sensitivity linear equations were largely consistent. At a depth of 0.2 mm the linearity of the sensitivity equation was relatively low, at 0.94, while at depths greater than 0.4 mm the linearity of the sensitivity equations was above 0.98, indicating good linearity. For microneedle electrodes with a total length of 1 mm, the penetration depth less than 0.2 mm significantly affected electrode sensitivity. This is likely due to friction and compression during the insertion process, which hinder the microneedle tip from fully contacting the ion environment, thereby impacting electrode performance. At depths greater than 0.4 mm, the sensitivity of the microneedle electrodes stabilized, and changes in penetration depth no longer affected electrode performance.

### 3.5. In Vivo Animal Experiments

The microneedle electrodes have been proven to be non-cytotoxic and exhibit good biocompatibility through biocompatibility testing (Figure 4e and Figure S16). The Cell Counting Kit-8 (CCK-8) test is a commonly used experimental method for assessing cell viability. In the experiment, 3T3 fibroblasts were revived, cultured, and passaged to obtain stable cultured cells. The microneedle reference electrodes and three types of ion-selective electrodes (potassium, sodium, calcium) were leached using serum-free culture medium to prepare the leachate. In the experimental group, the serum culture medium in the 24-well plates was replaced with the material leachate, while the control group maintained the same volume of liquid by adding serum-free culture medium. The OD values measured for different groups were recorded, and the average values were calculated to determine the corresponding cell viability for each group. The results, as shown in Figure 4e, indicated that during the three-day culture period, the cell viability using the leachate from the four types of microneedle electrodes remained above 80%. This demonstrates that these four types of microneedle electrodes exhibited no significant toxicity to the cells over three days, showing excellent biocompatibility. We conducted in vivo experiments using healthy SD rats, as shown in Figure 4b. To induce changes in electrolyte concentrations within the animals, we injected a 6% NaCl solution (0.5 mL/100 g) into the rats’ abdominal cavities. We used data from the first blood sampling point combined with monitoring system data to calibrate the sensitivity curves obtained from testing the electrodes in standard solutions. Using the calibrated sensitivity curves, we calculated the concentration values corresponding to the data collected by the monitoring system and compared them with standard concentration values, as shown in Figure 4f. Upon injecting a high concentration of NaCl solution, the sodium ion concentration in the rats’ bodies increased sharply. Due to the rapid permeation of the NaCl solution, the concentration changes became evident within 15 min post injection. Sodium ion concentration rose from 138 mM to 158 mM. Subsequently, the body began to excrete excess sodium ions through the kidneys, gradually lowering the sodium ion concentration back to normal levels, stabilizing at around 141 mM. As sodium ion concentration increased due to the injected solution, potassium levels in the blood decreased. Potassium ion changes typically occur more slowly and gradually, becoming noticeable about 30 min after injection. In our experiment, potassium ion concentration decreased from 4.9 mM to approximately 4.8 mM and eventually returned to 5.1 mM. Due to different regulatory mechanisms for sodium and calcium ions, calcium ion concentrations did not show significant changes, fluctuating between 2.4 mM and 2.5 mM. The test results demonstrated that the monitoring system has the advantage of real-time concentration level acquisition. The observed trends in concentration changes were consistent with those expected from standard tests, validating the feasibility and reliability of the monitoring system.

## 4. Conclusions

In this study, we developed a multi-parameter ion monitoring system based on microneedle arrays for the real-time continuous detection of subcutaneous ions. The research validated the excellent performance of ion-selective microneedle electrodes in terms of sensitivity, linear range, selectivity, and stability. Meanwhile, the reference electrode demonstrated outstanding selectivity for chloride ions and excellent stability, ensuring the reliability of the monitoring system under various conditions. Using finite element simulation methods, we analyzed the mechanical performance of microneedle arrays with different structural designs during skin insertion. Experimental validation confirmed the accuracy of the simulation results, demonstrating that increasing the spacing of microneedle arrays and optimizing their design can effectively reduce insertion force, improve insertion efficiency, and minimize skin damage. We employed an optimized integration process using flexible circuit substrates, making the assembly of microneedle arrays more convenient, and minimizing damage to the microneedle electrodes during assembly. By incorporating serpentine wiring and hollow array designs, we enhanced the flexibility of the array, allowing it to better conform to skin surface deformations. This improved flexibility enhances the applicability and comfort of the microneedle array in practical applications. A comprehensive multi-parameter ion real-time monitoring system was developed, comprising microneedle arrays, hardware circuits, and an upper-level computer system. This system enables the real-time monitoring of multiple ion concentrations in interstitial fluid. Extensive testing confirmed the system’s stability and feasibility. Through in vitro simulation experiments and in vivo animal tests, we demonstrated that the microneedle electrodes meet biocompatibility requirements and can stably monitor changes in ion concentrations within the body. These findings indicate that the technology has promising applications in clinical electrolyte monitoring.

## Data Availability

The data presented in this study are available on request from the corresponding author.

## Supplementary Materials

The following supporting information can be downloaded at: https://www.mdpi.com/article/10.3390/bios15050310/s1, Figure S1: SEM of microneedle electrodes with different etching times; Figure S2: EDS of the microneedle electrodes before and after reconstruction; Figure S3: The images of the ion-selective electrode membrane layer on the microneedle with and without reconstruction; Figure S4: CV scans of the microneedle electrode before and after reconstruction; Figure S5: SEM images of ion-selective microneedle electrodes prepared by three different film coating methods; Figure S6: stability of ion-selective electrodes; Figure S7: Performance of the reference electrode; Figure S8: Modeling of stainless steel microneedles; Figure S9: Finite element modeling simulation; Figure S10: Stretching and twisting deformations; Figure S11: The sensitivities of the assembled ion-selective electrodes; Figure S12: hardware circuit schematics; Figure S13: Flow chart of the application software; Figure S14: Operation interface of the application; Figure S15: In vitro simulation experiments; Figure S16: Biocompatibility experiments of the microneedle electrodes.
