# Supplementary material for: Individually Modified Microneedle Array for Minimal Invasive Multi-Electrolyte Monitoring
Source: Biosensors (Basel). 2025 May 12;15(5):310. doi: 10.3390/bios15050310 (PMC12110280; doi:10.3390/bios15050310)
Supplement: Supplementary file 1 [file biosensors-15-00310-s001.zip › biosensors-3617047-supplementary.pdf]

Supplemental Material

Individually Modified Microneedle Array for Minimal Invasive Multi-Electrolyte Monitoring

Ketian Yu[1a], *Yukun Ma*[1a], *Yiming Wei*[a], *Wanying Chen*[a], *Zhen Dai*[a], *Yu Cai*[b], *Xuesong Ye*[a] *and Bo Liang*[a,b]*

[a] Biosensor National Special Laboratory, Key Laboratory of Biomedical Engineering of Ministry of Education, College of Biomedical Engineering and Instrument Science, Zhejiang University, Hangzhou, Zhejiang 310027, PR China

[b] Binjiang Institute of Zhejiang University, Hangzhou 310053, PR China

[1]These authors contributed equally to this work.

*Correspondence: boliang1986@zju.edu.cn (B. Liang)

S1: Preparation of Ion-Selective Electrodes Based on Microneedles

Electrode Preparation Reagents and Instruments: Reagents: Sodium ionophore X, dioctyl sebacate (DOS), sodium tetra[3,5-bis(trifluoromethyl)phenyl]borate (Na-TFPB), potassium tetrakis(4-chlorophenyl)borate (KTClPB), 3,4-ethylenedioxythiophene (EDOT), poly(sodium 4-styrenesulfonate) (NaPSS), polyurethane (PU), and gold etchant were purchased from Sigma-Aldrich. Valinomycin and calcium ionophore IV were purchased from Aladdin (Shanghai, China). Tetrahydrofuran (THF), sodium chloride, potassium chloride, calcium chloride, magnesium chloride, hydrochloric acid, etc., were all purchased from Guoyao Reagent Co., Ltd. Gold-coated microneedle electrodes and silver-coated microneedle electrodes were purchased from Baiyi Technology Co., Ltd. Solutions were prepared using 18 MΩ • cm (at 25° C) deionized water (DI). Instruments: Electrochemical workstation (μAutoLab III, Metrohm, Switzerland), Ultrasonic cleaner (Xiao Mei Ultrasonic Instrument, model XM-P102H), Magnetic stirrer, Oven, Electronic balance.

Preparation of PEDOT: PSS Electroplating Solution: Use deionized water as the solvent and purge with nitrogen for 30 minutes to remove oxygen from the water. Sequentially add 0.01 M EDOT and 0.1 M NaPSS to the deionized water, shake until fully dissolved, and then use ultrasonication to remove any air bubbles in the solution.

Preparation of ISM Solution: The composition of the ISM membrane solution is shown in Table S1. When preparing the ISM solution, first dissolve the ionophore, ion exchanger, and plasticizer sequentially into 1 ml of THF solvent; after thorough dissolution, add the polymer matrix and shake until it is completely dissolved.

Preparation of the Reference Solution: Dissolve 395.5 mg of PVB in 5 ml of methanol to prepare a 10% wt. PVB solution. Seal and store the well-mixed solution in a refrigerator at 4° C to prevent solvent evaporation. Weigh 50 mg of NaCl and 50 mg of $AgNO_3$, and dissolve them in 1 ml of the above-prepared PVB solution. After ultrasonic treatment, the solution appears milky white due to the low solubility of

AgCl in methanol, resulting in numerous AgCl particles in the solution. Expose the obtained milky white solution to white light, and observe that the solution changes from white to light purple. This color change is due to some of the AgCl being reduced to metallic Ag in the solution, altering the colloid's color. The prepared light purple solution is the reference electrode solution, which should be stored away from light.

Table S1 Composition of ISM solution

Ion	Ionophore/mg			Ion exchanger/mg		Plasticizer/mg	Polymer matrix/mg
	Valinomycin	Sodium Ionophore X	Calcium Ionophore IV	NaTFPB	KTClPB	DOS	PU
K^+	2.00	0	0	0	0.55	66	33
Na^+	0	1.00	0	0.55	0	66	33
Ca^{2+}	0	0	1.00	0.90	0	66	33

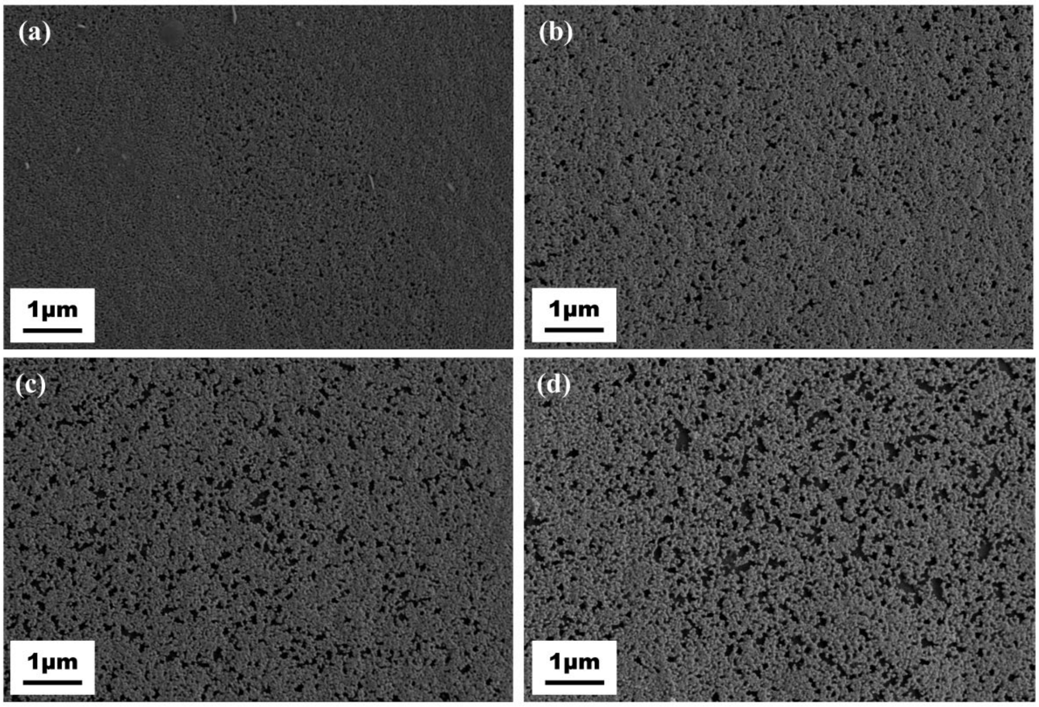

Figure S1: During the pre-treatment of the microneedle substrate, scanning electron microscopy (SEM) tests were conducted on microneedle electrodes that were immersed in an etching solution for 60 s, 120 s, 180 s, and 300 s(a, b, c and d). From the images, it can be observed that the microneedle electrode immersed for 60 s shows a relatively shallow reconstruction with only a few small holes appearing, and the surface remains basically smooth; for the microneedle electrode immersed for 120 s, a noticeable rough structure can be observed on the surface, and the texture is uniform; the microneedle electrodes immersed for 180 s and 300 s exhibit a large number of holes on the surface, with significant etching of the gold coating layer, leading to structural damage. Therefore, we chose 120 s as the optimal immersion time to ensure a rough surface structure on the microneedle electrodes while preserving their overall integrity without causing structural damage.

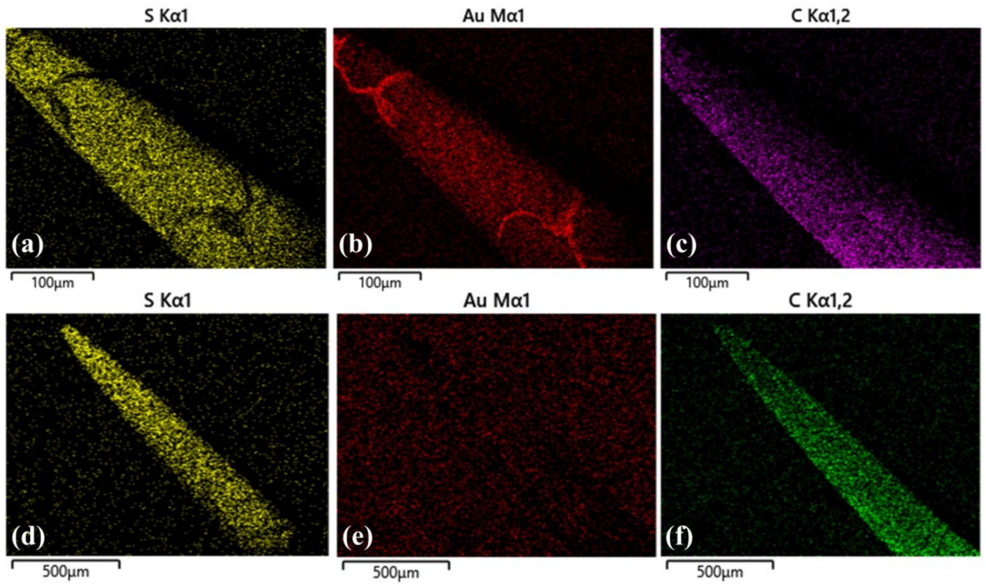

Figure S2: Since sulfur (S) is a characteristic element of PEDOT:PSS, energy-dispersive spectroscopy (EDS) was used to characterize the microneedle electrodes before and after reconstruction. This allows for a more in-depth analysis of the locations where cracking occurs. The EDS scanning results indicate that on the electrode without substrate reconstruction(a, b and c) cracking is observed in the PEDOT:PSS layer of the microneedle electrode (yellow portion in (a)). In the cracks, the underlying gold coating layer can be seen (red-highlighted area in (b)), indicating that the gold coating layer adheres strongly to the microneedle substrate and does not crack. On the electrode with substrate reconstruction(d, e and f), the surface of the reconstructed microneedle electrode is intact and shows no signs of cracking.

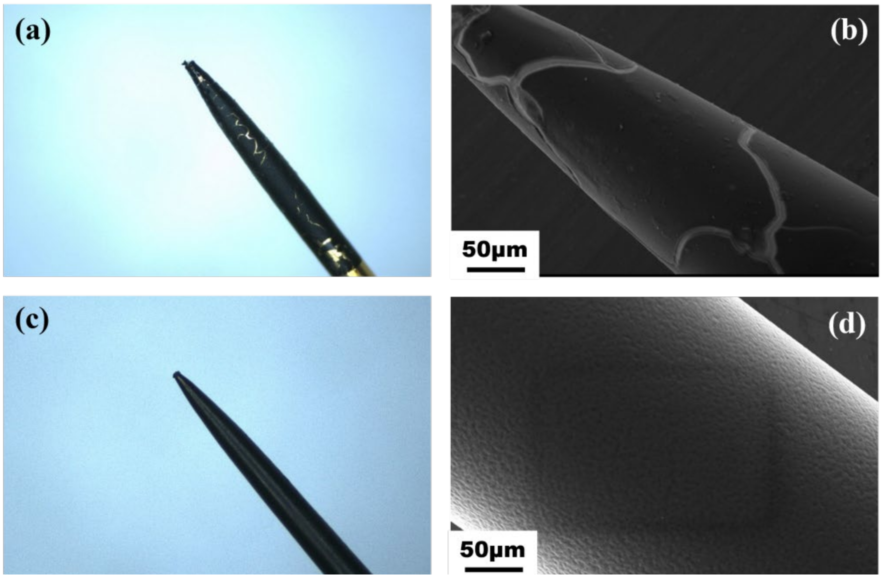

Figure S3: The robustness of the ion-selective electrode membrane layer on the reconstructed microneedle has significantly improved compared to the microneedle without reconstruction. Before reconstruction(a and b), due to the smooth and flat surface of the microneedle, the electro-polymerized PEDOT:PSS did not adhere firmly to the substrate. When the coated ion-selective membrane (ISM) layer dried, the stress imbalance between the ISM layer and the PEDOT:PSS layer caused cracks in the PEDOT:PSS layer. After reconstruction(c and d), the microneedle surface became rougher, leading to a stronger adhesion between the PEDOT:PSS layer and the substrate. As a result, no cracking occurs after coating with the ISM layer.

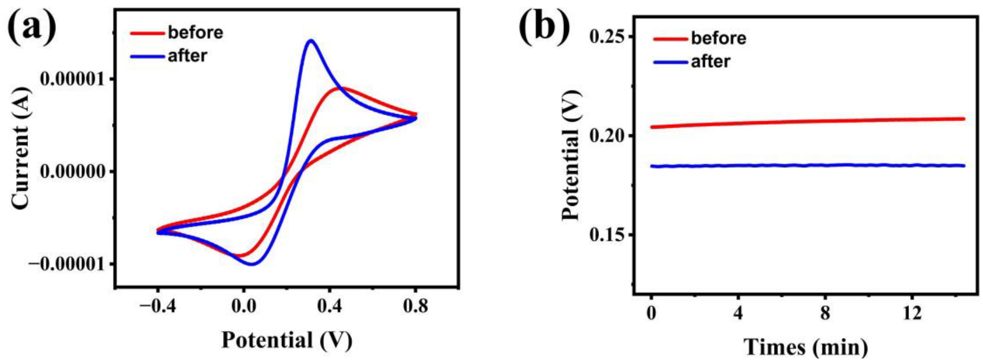

Figure S4: (a) In a solution containing 10 mM/L $K_3Fe(CN)_6$/$K_4Fe(CN)_6$ and 0.1 M KCl, cyclic voltammetry (CV) scans were performed on the same microneedle electrode before and after etching for 120 seconds at a scan rate of 50 mV/s. From the figure, it can be observed that compared to the untreated electrode, the treated electrode shows a larger current area. This is due to the electrode surface becoming rougher, which results in a greater specific surface area for the reconstructed microneedle electrode, providing more active sites for reactions, thereby enhancing the chemical response. Additionally, the oxidation peak shifts negatively, and the reduction peak shifts positively, indicating that the electrode becomes more easily oxidized and reduced. (b) Compared to the microneedle electrode before reconstruction, the microneedle electrode after reconstruction exhibits better stability. Both types of electrodes were placed in target ion solutions of the same concentration for long-term testing. The sensitivity signal of the microneedle electrode before reconstruction shows a certain trend of change over time, approximately 16.45 mV/h. The change rate of the microneedle electrode after reconstruction is 1.68 mV/h, indicating superior electrode stability. This is because the electrode before reconstruction, due to surface film cracking, is more likely to form a water layer between the film and the electrode during prolonged soaking in the solution, which greatly affects the stability and lifespan of the electrode.

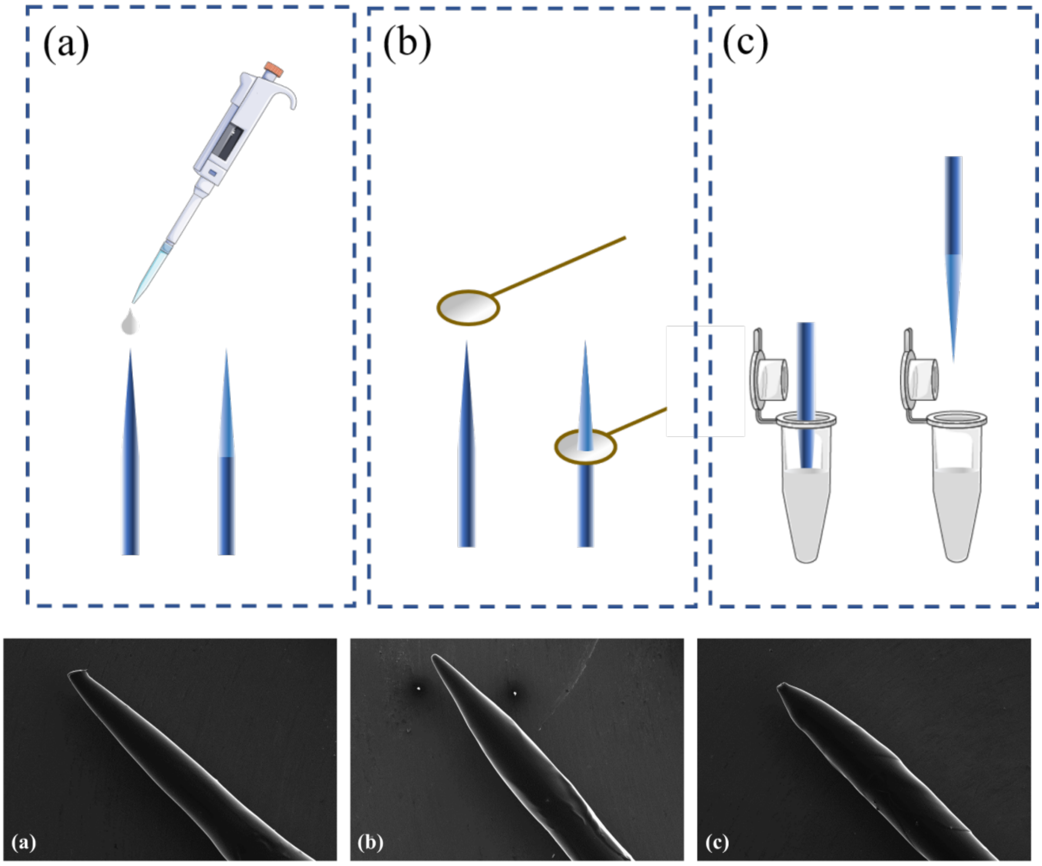

Figure S5: We tried the three methods shown in the figure to modify the microneedle electrodes. (a) Dipping Method: using a pipette to draw a fixed volume of ISM solution and then dripping it onto the tip of the microneedle, allowing the ISM solution to naturally flow down the surface of the microneedle to form a thin film. (b) Ring Coating Method: using a copper ring to dip into the ISM solution, forming a liquid film within the copper ring, and then passing the microneedle through the copper ring, which can result in a uniform film on the surface of the microneedle. (c) Dip Coating Method: immersing the microneedle in the ISM solution and then withdrawing it at a constant speed, forming a thin film on the surface of the microneedle; once the THF in the ISM solution has completely evaporated, a solid film forms on the surface of the microneedle. The SEM images below show the morphology of ion-selective microneedle electrodes prepared by three different film coating methods. As observed from the electron microscope results, the film layer of the microneedle electrode prepared by the ring coating method is more uniformly

covered; although the dipping method is widely used for planar electrodes, it is less effective for microneedle electrodes, for the film solution has strong adhesion and the solvent evaporates quickly, making it difficult for the film solution to smoothly cover the entire surface of the microneedle electrode, resulting in uneven film layer modification; for the dip coating method, the film solution tends to accumulate at the tip of the microneedle, resulting in an uneven surface at the tip of the microneedle electrode.

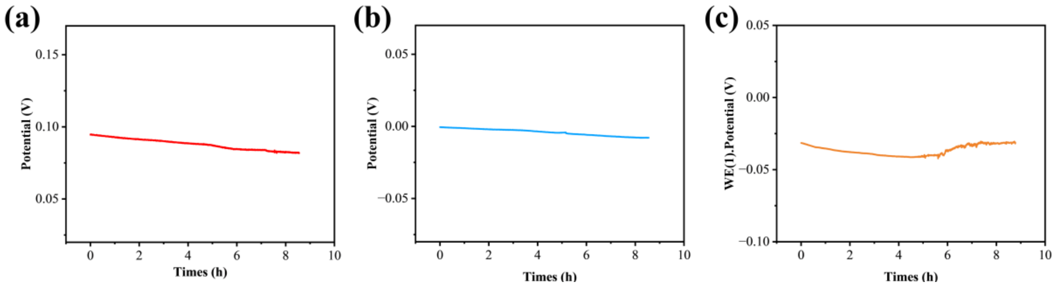

Figure S6: Testing the long-term stability of ion-selective electrodes. The ion-selective microneedle was immersed in a solution containing the target ions, and its potential change was tested overnight. The potential change rate of the ion-selective electrodes is (a)potassium 1.5 mV/h, (b)sodium 0.9 mV/h, (c)calcium 0.7 mV/h. These results indicate that the prepared ion-selective electrodes have good stability.

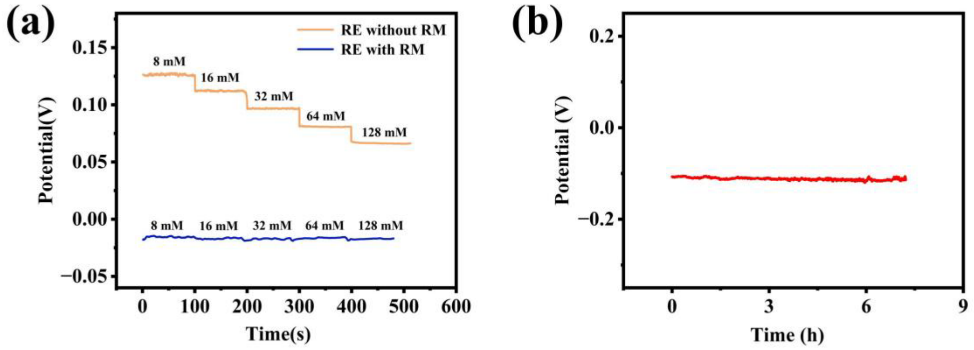

Figure S7: Performance of the reference electrode. (a) Based on the preparation of Ag/AgCl electrodes by chloridizing silver-coated microneedles, we modified the reference electrode film layer and compared it with a reference electrode coated with commercial Ag/AgCl paste. The two types of electrodes were tested in NaCl solutions of different concentrations. The potential of the electrode coated only with Ag/AgCl paste decreases as the chloride ion concentration increases, showing a pattern that conforms to the Nernst equation; in contrast, the microneedle electrode coated with the reference electrode solution is not affected by chloride ion concentration and maintains a stable potential in solutions of different concentrations, making it an ideal microneedle reference electrode. (b) The potential change of the microneedle reference electrode was tested overnight in a 0.1 M NaCl solution. The potential change rate of the reference electrode is 0.9 mV/h, indicating good stability.

S2: Finite Element Modeling Simulation and Experimental Validation

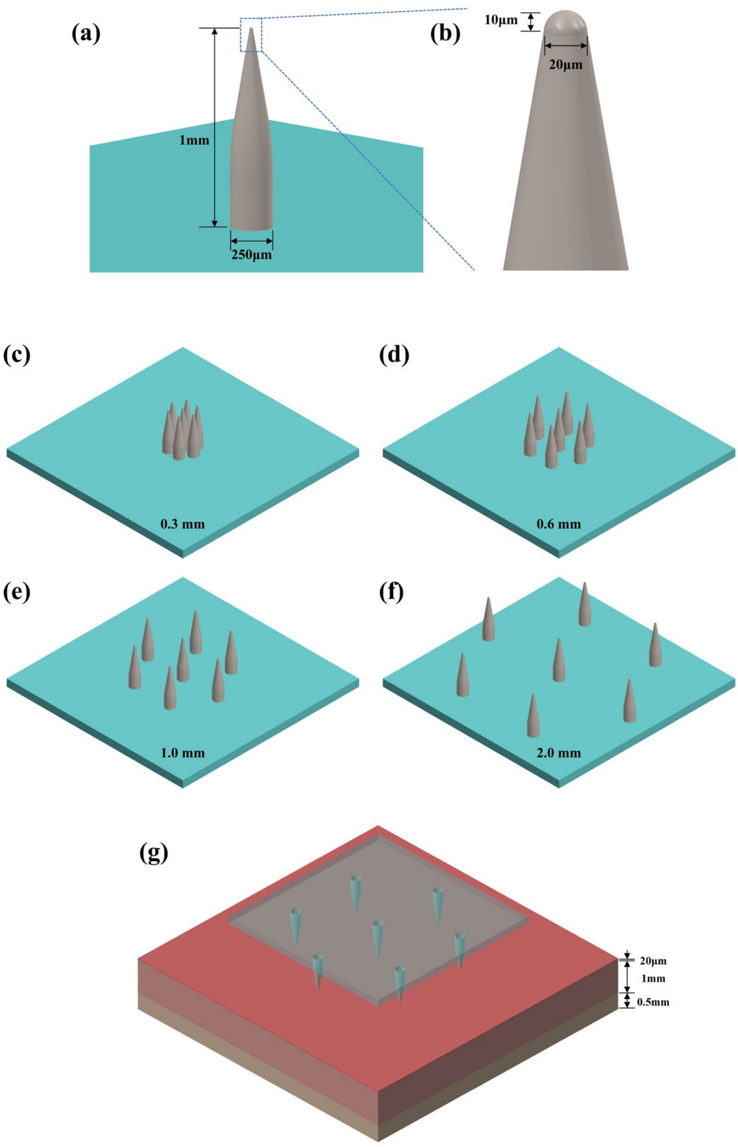

Figure S8: (a,b) Modeling stainless steel microneedles. The stainless-steel microneedle is a two-segment type, consisting of an upper conical section and a lower cylindrical section. The transition between the conical and cylindrical sections is smooth. In the microneedle modeling, a substrate at the bottom of the microneedle (the blue-green part) was designed to simulate the substrate of the microneedle array patch used in applications.

(c,d,e,f) Based on the geometric parameters of a single microneedle, circular microneedle arrays with different spacings were designed to simulate real-world conditions and guide subsequent designs. The circular microneedle arrays are centered

around a central microneedle, with radii of 0.3 mm, 0.4 mm, 0.6 mm, 0.8 mm, 1 mm, 1.5 mm, and 2 mm. Six microneedles are evenly distributed at each radius. The needle body of the microneedle array has the material properties of stainless steel, with a density of 7.9 g/cm³, Young's modulus of 20900 MPa, and Poisson's ratio of 0.3.

(g) In many studies, due to computational cost constraints, the entire skin is modeled as a single entity, ignoring the multi-layer structure of skin tissue. In this study, based on actual skin tissue, a multi-layer skin model was designed, consisting of the stratum corneum, dermis, and subcutaneous tissue. The skin tissue model is an 8 mm * 8 mm * 1.52 mm rectangular prism, with the contact surface size with the microneedle set to 8 mm * 8 mm, which is much larger than the contact area of the microneedle array, thus avoiding the influence of boundary conditions of the skin tissue on the simulation results. Skin tissue is anisotropic, nonlinear, and hyperelastic material. During modeling, different mechanical models and material parameters were set for the stratum corneum, dermis, and subcutaneous tissue. The hyperelastic Neo-Hookean model was used to describe the nonlinear behavior of multiple skin layers during microneedle insertion, since it does not rely on linear relationships between stress and strain but instead use strain energy density functions to describe material behavior, making them suitable for modeling soft materials like biological tissues

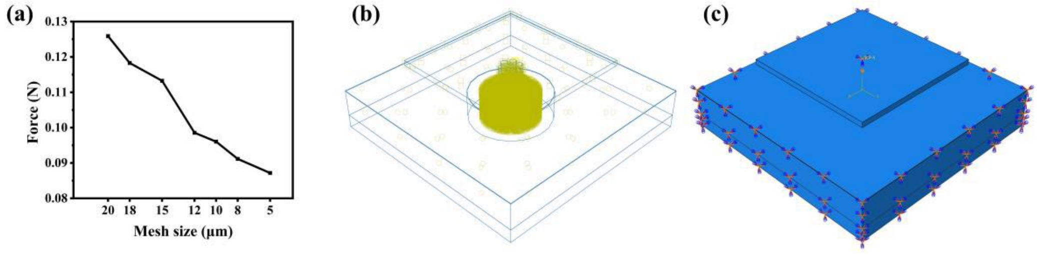

Figure S9: (a) In finite element simulations, meshing is a critical factor that affects both the accuracy of the simulation results and computational efficiency. The rationality of meshing directly impacts the accuracy of the mechanical response and contact between microneedles and skin tissue. For microneedle arrays, which have much higher stiffness compared to skin tissue, they can be considered as rigid bodies. Given the small and intricate structure of microneedles, tetrahedral meshes are chosen for meshing the microneedle array. For multi-layered skin tissue, since the puncture site will experience greater stress and deformation during the simulation, non-uniform meshing is applied to the skin tissue. Fine meshes are set in the contact area between the microneedles and the skin. Hexahedral meshes are selected for the skin tissue to ensure grid quality during large deformations, thus providing higher accuracy and stability in the simulation calculations. To compare the puncture force under different mesh sizes, mesh convergence analysis was performed. The study sizes selected were 20 μm, 18 μm, 15 μm, 12 μm, 10 μm, 8 μm, and 5 μm. The results showed that when the mesh size was refined to 5 μm, the calculation results gradually converged. Therefore, 5 μm was chosen as the appropriate mesh size.

(b,c) In the contact settings, constraints are applied to the simulation model, and the contact and friction generated during the microneedle insertion process are configured. During the simulation, due to the much higher rigidity of the microneedles compared to skin tissue, a rigid body constraint is applied to the microneedles, and force analysis is performed on the reference point of the rigid constraint. TIE constraints are applied to the contact surfaces of different layers of skin tissue to prevent separation between layers. During the microneedle insertion into the skin, the microneedle first

contacts the skin surface. When the stress on the surface elements exceeds the failure stress, these surface elements fail, allowing the microneedle to come into contact and friction with the internal skin tissue elements. To achieve this effect, surface-to-surface contact is set between the microneedle and the internal skin tissue. The primary surface is the microneedle array surface, and the secondary surface type is nodes, including the node sets of skin tissue elements that contact the microneedle. In boundary conditions, a displacement of 1 mm is applied to the microneedle array via the reference point of the rigid constraint, ensuring the microneedle penetrates exactly into the skin. Fixed constraints are applied to the outer surfaces of the skin tissue (except for the surfaces in contact with the microneedle) to prevent any displacement. The simulation process is analyzed using Abaqus/Explicit (explicit solving algorithm), which is suitable for simulating rapid dynamic processes over short periods. In the mass scaling option, by controlling the size of the time step, the computational efficiency of the system is improved, optimizing computational convergence.

S3: Assembly of microneedle array and design of the monitoring system

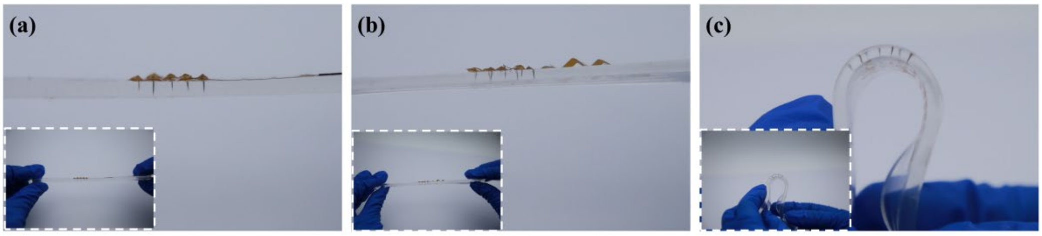

Figure S10: To verify that the optimized array can withstand stretching and twisting deformations on the epidermis, polydimethylsiloxane (PDMS) was prepared to simulate skin. To ensure that PDMS has sufficient elasticity, the ratio of PDMS to curing agent was adjusted to 40:1. The microneedle array was fully inserted into the PDMS, and stretching and twisting tests were performed. When the PDMS is stretched or twisted, the array can deform along with the PDMS, meanwhile, the microneedles remain securely embedded in the PDMS without detaching from the simulated skin.

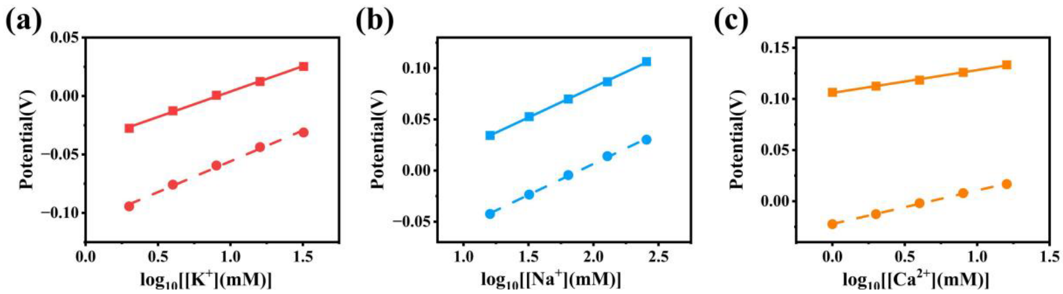

Figure S11: Before assembling the array, the sensitivity of the working electrodes was tested using a standard reference electrode. The results are shown as solid lines in the figure, while the sensitivities after assembly are shown as dashed lines. The sensitivities of the assembled ion-selective electrodes are as follows: Potassium 52.69 mV/decade, Sodium 60.71 mV/decade, Calcium 32.85 mV/decade. These values conform to the Nernst equation. The overall shift in potential before and after assembly is due to the potential difference between the microneedle reference electrode and the standard reference electrode, which does not affect the performance of the ion-selective electrodes.

Table S2 Key parameters of MAX9912

Parameter	Minimum value	Typical value	Maximum value	Unit
Operating Voltage	1.8	-	5.5	V
Power consumption	-	7	-	μA
Input bias current	-	±1	±10	pA
Input offset voltage	-	±0.2	±1	mV
Input impedance	-	1	-	GΩ

Table S2: The working environment of the microneedle array is human tissue fluid, so the selection of operational amplifiers in the backend detection circuit is a crucial step to ensure the accuracy and stability of electrochemical measurements. For this working environment, the required operational amplifiers need to have the following characteristics: low input bias voltage and low input offset current, low noise performance, high input impedance, low power consumption. The MAX9912 chip from Analog Devices is a low-bias current operational amplifier that features extremely low power consumption, low bias current, low offset voltage, and high input impedance. It meets the requirements of the electrode system and also provides multiple package options for portable wearable devices, making it an ideal choice for designing the detection circuit.

When designing portable wearable devices, the selection of the main control chip determines the system performance and user experience. Choosing a main control chip with lower power consumption, higher integration, and smaller packaging is beneficial for developing more accurate and multi-functional detection systems. The

low-power Bluetooth (Bluetooth Low Energy, BLE) wireless microcontroller CC2640 introduced by Texas Instruments (TI) has these advantages, featuring built-in high-resolution analog-to-digital converters (ADCs) and other peripheral interfaces. It is very suitable for use in wearable devices, Internet of Things (IoT), and other wireless communication applications. Therefore, this design selects CC2640 as the main control chip.

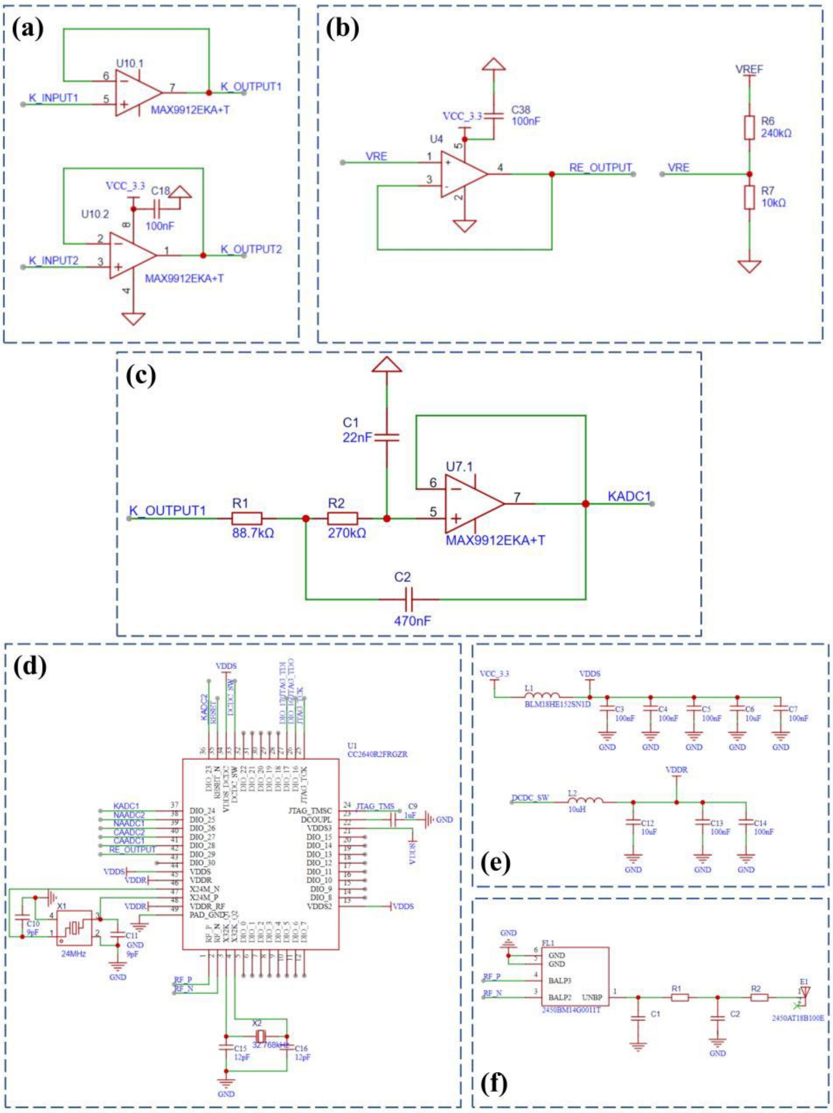

Figure S12: (a,b) The analog front end is composed of voltage followers. The working electrode and reference electrode are connected to the input terminals of the voltage follower. To adjust the measurement potential range, a voltage divider circuit is used to apply a reference voltage to the reference electrode. The figure shows two potassium ion electrode channels, where K_INPUT is connected to the working electrode, and K_OUTPUT outputs the potential of the working electrode. VRE is connected to the reference electrode to apply the reference voltage, and RE_OUTPUT outputs the potential of the reference electrode.

(c) To ensure the stability and accuracy of the measurement signals, a second-order filter is set up to eliminate various environmental noise interferences, such as power

frequency interference. A Sallen-Key second-order low-pass filter circuit is designed, with all resistor and capacitor parameters determined by calculation.

(d,e,f) Main control circuit. After the potential signals are filtered, they are connected to the ADC port of the main control chip. Through internal program control, the main control chip can continuously collect the potential signals of the electrodes and generate the open circuit potential (OCP) curve of the electrode. In the figure is the circuit diagram of the CC2640 main control system, where the filtered potential signals of each electrode are connected to the ADC port of the CC2640. The 2450BM14G0011T balun chip and the 2450AT18B100E antenna are selected to form the RF circuit, which transmits data in real-time to the upper computer system via low-power Bluetooth.

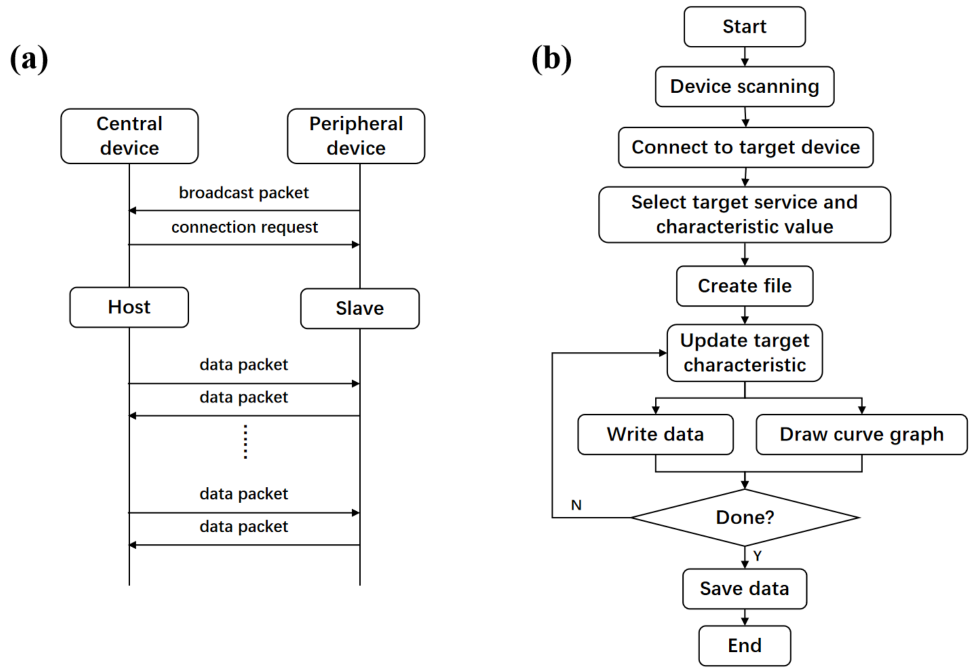

Figure S13: To facilitate data analysis, we have designed an application software called E-Monitor for connecting hardware devices and performing real-time data acquisition, processing, and display. (a) After the peripheral device is activated, it sends broadcast packets through the broadcast channel, containing device information. The central device listens to the broadcast channel and, upon receiving the broadcast packet from the peripheral device, sends a connection request to the peripheral device. Once the connection is established, both parties communicate over the data channel as the host and slave, respectively. (b) After opening the application, the mobile phone, acting as a central device, begins scanning for peripheral Bluetooth devices and activates the hardware detection circuit. Users can discover and connect to the detection device. Upon successful connection, services and characteristic values of the Bluetooth device can be read. The characteristic values contain data packets such as potentials sent by the detection circuit. These data packets are updated every second according to the program settings. Create a file in the root directory to record the data. When the data packet is updated, the data is written into the file, and the curve is plotted and updated within the program. To improve data writing efficiency, a

buffering mechanism is employed, where data is collected in batches of 60 entries before being written to a CSV file at once.

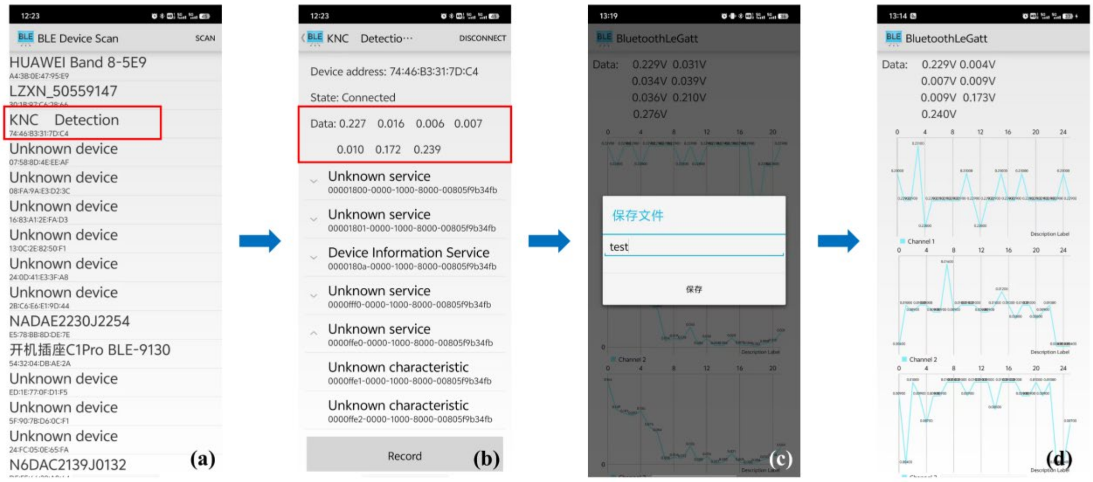

Figure S14: Operation interface of the application. (a) Open the application and select the "KNC Detection" device to connect. (b) After the hardware device is successfully connected, "Connected" is displayed in the State bar. At this point, select the target service (Service) and characteristic value (Characteristic). The data packets corresponding to the selected characteristic will be displayed in the Data bar. Click the "Record" button at the bottom of the interface to start recording data. (c) Enter the recording interface. In the pop-up dialog box, input the file name and click the "Save" button to save the file. Subsequently, data packets are continuously written into this file. (d) Continuously receive data packets and plot the curve for each channel.

S4: In Vitro Simulation Experiments

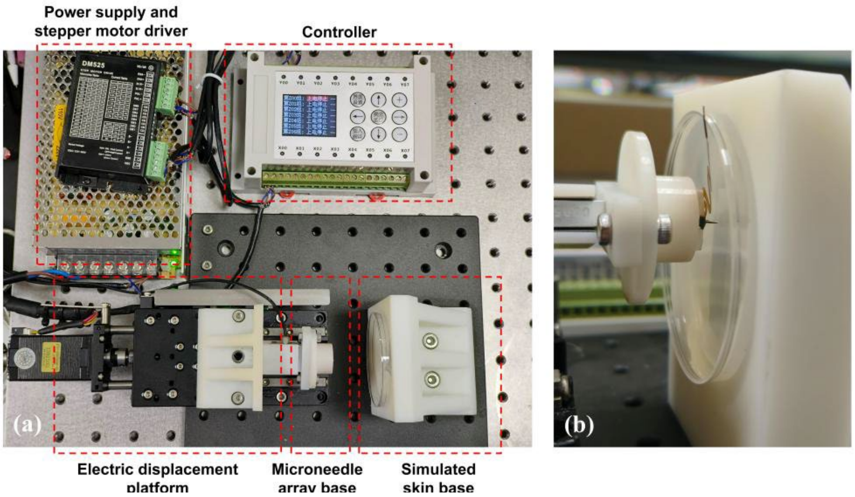

Figure S15: To precisely control the penetration depth of microneedle arrays, a testing platform was constructed using an electric displacement stage. The range of this electric displacement stage is 250 mm, with a precision that can reach 5 μm. The electric displacement stage is controlled by a stepper motor driver. During use, the pulse frequency and number of pulses are controlled via the controller to determine the speed and distance of each movement of the electric displacement stage. A base for the microneedle array was designed and printed, then fixed onto the moving platform of the electric displacement stage. Additionally, a base for simulating skin was designed and printed at one end of the moving axis of the electric displacement stage, used to secure the simulated skin. Agarose gels at 1% w/v were prepared using NaCl solutions of different concentrations to simulate skinns with interstitium fluid containing different ion concentrations. During testing, controlling the movement of the electric displacement stage allows precise control over the penetration depth of the microneedle array.

S5: Biocompatibility Experiments

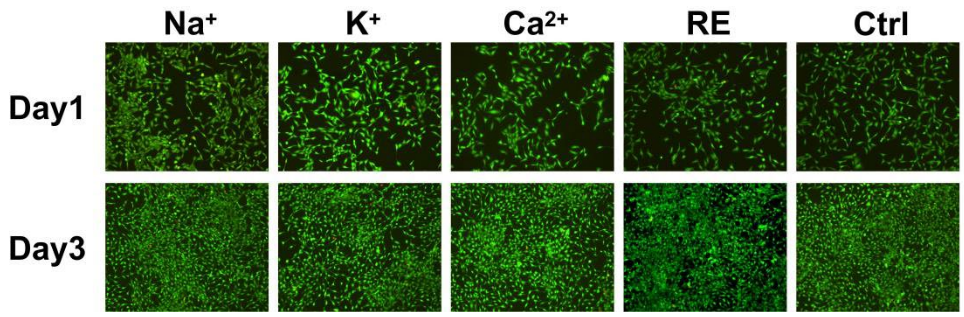

Figure S16: 3T3 fibroblasts were seeded at the same and appropriate density in cell culture dishes. Each group was treated with the leachate of reference electrodes and three types of ion-selective electrodes (experimental groups) or serum-free medium (control group). The cells were cultured for three days. The cells in each group were stained using a Calcein/PI kit and observed under a microscope. Only a very small number of dead cells, which appeared red, were observed within the field of view, and the cell density increased over the three days, demonstrating that the microneedle electrodes are non-toxic to cells and have good biocompatibility.
